# Stimuli-Responsive Zinc (II) Coordination Polymers: A Novel Platform for Supramolecular Chromic Smart Tools

**DOI:** 10.3390/polym13213712

**Published:** 2021-10-27

**Authors:** Rosita Diana, Ugo Caruso, Barbara Panunzi

**Affiliations:** 1 Department of Agricultural Sciences, University of Naples Federico II, 80055 Portici, Italy; rosita.diana@unina.it; 2 Department of Chemical Science, University of Naples Federico II, 80126 Napoli, Italy; ugo.caruso@unina.it

**Keywords:** zinc (II) coordination polymer, luminescence, sensing, bioimaging, smart tools

## Abstract

The unique role of the zinc (II) cation prompted us to cut a cross-section of the large and complex topic of the stimuli-responsive coordination polymers (CPs). Due to its flexible coordination environment and geometries, easiness of coordination–decoordination equilibria, “optically innocent” ability to “clip” the ligands in emissive architectures, non-toxicity and sustainability, the zinc (II) cation is a good candidate for building supramolecular smart tools. The review summarizes the recent achievements of zinc-based CPs as stimuli-responsive materials able to provide a chromic response. An overview of the past five years has been organised, encompassing 1, 2 and 3D responsive zinc-based CPs; specifically zinc-based metallorganic frameworks and zinc-based nanosized polymeric probes. The most relevant examples were collected following a consequential and progressive approach, referring to the structure–responsiveness relationship, the sensing mechanisms, the analytes and/or parameters detected. Finally, applications of highly bioengineered Zn-CPs for advanced imaging technique have been discussed.

## 1. Introduction

### 1.1. Brief Historical Background

The work of Alfred Werner (Nobel Prize for Chemistry in 1913) and his contemporaries laid the foundation for the study of coordination polymers (CPs). The first preparation and application of coordination polymers traces back as far as early 18th century, when German chemists accidentally discovered the Prussian blue dye (empirical formula Fe_4_[Fe(CN)_6_]_3_). Crystallographic analysis revealed Prussian blue to be a 3D coordination polymer where Fe^2+^ cations are coordinated by the carbon atom of the cyanide ligand that acts as a bridge with Fe^3+^ cations lying in an octahedral N-coordination core. In 1967, J.C. Bailer first introduced the term “coordination polymer”, establishing the constructional requirements and characteristic properties of the CPs as hybrid organic–inorganic materials. More recently, significant advances were due to Professor Richard Robson of the University of Melbourne, who published over 200 articles on coordination polymers and is considered “a pioneer in crystal engineering involving transition metals“ [1,2]. Specifically, Robson underlined the unicity of the 3D CPs, where the organic ligands extend up to infinity thanks to the coordination bond even in addition to other non-covalent weak interactions. As a pioneer, Robson perceived the structural uniqueness of the regular tridimensional CPs, which today are considered the “frontier” of hybrid responsive materials.

Many time-honoured materials are now recognized as coordination polymers. From the first work published in 1997 [3] to recent years, there are more than 4000 works focusing on specific photophysical properties and/or applications of the coordination polymers (CPs). 

### 1.2. The Role of the Coordination Bond in the Supramolecular Polymers

The polymers are long-chain molecules (macromolecules) built from individual molecular blocks held together via covalent bonds. Where the covalent bond ends, supramolecular materials begin. Supramolecular chemistry concerns non-covalent interactions between small molecules able to self-assemble into macromolecular aggregates. Di-topic or poly-topic ligands with two or more interactive sites can be employed. The supramolecular interactions range from hydrogen-bonding to electrostatic interactions and coordination bonds. The non-covalent “weak” interactions throughout the frameworks direct the self-assembly of mono, bi or three-dimensional (abbreviated as 1D, 2D or 3D) macrostructures [4,5].

The formation of the coordination bond is the key factor in an eminent number of supramolecular aggregates. In a coordination compound, donor ligands share their electron pairs with a metal atom which is commonly a transition metal cation. Translating this simplified concept to polymers, the coordination polymers (CPs) are solid-state structures consisting of 1, 2 or 3D repeating coordination units arranged in an ordered or not-ordered structural habitus [6,7,8,9]. 

Coordination polymers in which metal ions and organic ligands are linked into 3D structures with a specific nano- (<2 nm) or meso- (2–50 nm) porous structure resulting from a tridimensional crystalline regularity are often referred as metal-organic frameworks (MOFs). Such functional cavities can be target designed for a selective interaction or entrapment, so that MOFs encompass one of the most sought-after class of hybrid responsive materials [10,11,12,13,14,15]. The properties of the supramolecular CPs are based on the association and functionality of the organic building blocks and/or the metal nodes. Several factors play a role towards the engineering of targeted CPs, including association, shape, size, functionality, and connectivity of building blocks. The dimensionality of the CPs depends on the organization of the building blocks, i.e., the organic ligands, the metal ions, the counter ions, and the solvent molecules. The functionality of the ligands is critical for the development of various weak interactions throughout the framework, which in turn are crucial for the construction and spatial arrangement of the material. 

An infinite possibility of structures and functions can be designed and realised to direct the interacting sites to target specific applications. In this context, theoretical analysis has constituted a growing support for the design of targeted structures [16,17,18,19,20,21,22,23,24]. The general idea is to exploit the properties of both the organic part and the inorganic part. Metals can conduct electricity and sometimes show magnetic properties. Metals are also catalysts for a wide range of reactions and undergo complexation–decomplexation equilibria under specific conditions. On the other hand, the organic ligands can provide useful optical properties and undergo intermolecular interactions with specific sites and/or analytes. If the sum of properties can be incorporated into a single hybrid material, it could have all sorts of applications. With the feature of high surface area and porosity, tuneable optical properties, adsorption affinity, magnetic properties, and CPs had been utilized in the field of gas storage [25,26,27,28,29,30,31,32,33,34,35] and separation [36,37,38], chemical sensing [39,40,41,42,43], energy conversion [44,45,46], heterogeneous catalysis [47,48,49], gas and liquid purification and separation [50,51,52], drugs delivery [53,54,55,56,57], data storage and processing, and bioimaging [57,58].

### 1.3. General Considerations about Chromic Stimuli-Responsive CPs

Stimuli-responsive polymers are often referred to as smart polymers or (generally) functional materials. Their ability to change physical and/or chemical characteristics in response to external stimuli is the result of the specific composition and can be related to de-solvatation, ionization, crosslinking, and finally alteration of the inter-/intra-chains interactions [59,60,61,62,63,64,65,66,67]. It is obvious that CPs, as supramolecular self-assembled polymers, are “par excellence” responsive smart materials [68,69,70].

In such materials, thermodynamics and kinetics play complementary roles. Metal-ligand interactions can be thermodynamically stable but also kinetically labile, depending on the metal ion and ligand. The thermodynamic stability of the metal–ligand interactions will determine the degree of polymerization, the architecture, and the dynamics/lifetime of the coordination aggregate. On the other hand, the kinetic lability should result in a “dynamic” macrostructure in continual equilibrium, therefore, being potentially responsive [71]. CPs can easily respond to an extern stimulus via a dynamic dissociation/recombination equilibrium of non-covalent interactions. Smart functional CPs are attracting a growing interest within the study of life processes and technological applications. Their intrinsic properties make them ideal candidates for analytical and diagnostic dynamic applications. Stimuli-responsive CPs [12,72,73,74,75] can exhibit changes in a macroscopic property to a specific input, such as light, temperature, physical stress, solvent, analytes, electricity, ionic strength, and pH. The design of tailored stimuli-responsive CPs has attracted significant attention in the past decade due to their stimuli-responsiveness and self-healing ability [76,77,78,79].

As a new frontier in the smart materials research, here, we will focus attention to “chromic” materials. By chromic materials, we mean tools able to produce an optical signal within the visible spectrum detectable in absorbance (colorimetric response) and/or in emission (luminescence response) mode, as a response to an external stimulus. Materials able to emit fluorescence in the visible regions, enabling a chromic naked eye response are commonly referred as “fluorophores”. In addition to the obvious advantage to produce a naked eye appreciable signal [80], chromic tools can guarantee a real-time, low expansive, and in situ analysis. The high surface areas, tuneable porosity, and tailorable composition of the coordination polymers perfectly matches the requirements of chromic smart tools. According to the kind of stimulus that they respond to, examples of the major kinds of chromism are:

- Thermochromism, which is induced by a change of temperature;
- Mechanochromism, which is induced by a stress in the solid state;
- Electrochromism, which is induced by the gain and loss of electrons;
- Solvatochromism, which is induced by the polarity of different solvents;
- Photochromism, which is induced by light irradiation;
- Chemochromism, which is induced by specific chemical analytes.

Due to their wide responsiveness, chromic smart materials are now applied as sensors [61,81,82,83,84], safety devices [85], information and imaging technology [86,87,88,89,90,91], optoelectronic systems [92,93,94,95], smart windows and optical switches [96,97,98], inkless and erasable printing [99], and catalysis [100]. 

The versatile group of CPs provides an infinite number of candidates for the new generation of chromic smart materials, with potential applications both in the bulk state and at the molecular level. Particularly relevant is the photoluminescence (PL) performance which benefits from the tunable hybrid structure and electronic properties of CPs [74]. The general mechanism for luminescence in Cps involves in all cases the absorption of a photon (raising an electron from the ground to an excited state) and the subsequent return to the ground state with the emission of a photon [101]. However, the PL active channels involved in CPs’ emission can be complex and different for each case. Specifically, PL properties can be due to the organic part but also to host–guest interactions or interactions between interpenetrating frameworks. In addition, in CPs, the luminescence often originates from energy bands rather than from discrete energy levels. Finally, different metal cations can bring about radical changes in the PL pattern of the organic ligands. The possible excitations in transition metal complexes will be discussed in Section 1.3, with a specific focus on zinc (II) metal ion. 

PL properties may be critical to biological applications. A biosensor must include a biomolecular interaction able to give rise to an output signal. Biology and nanotechnology both inhabit the same length scale, so that nanosized probes are among the recent advanced tools for sensing of biological relevant entities and/or parameters in medical diagnostic. On the other hand, when materials are reduced to the nanoscale (5–200 nm), their properties can be dramatically altered respect to those observed in bulk. Miniaturization of CPs to the nanoscale offers a unique opportunity to assemble highly customizable functional materials. Responsive nanoscaled coordination polymers (NCPs) marry the tunability of the coordination complexes to the advantages of nanoprobes for an optimal use in sensing, recognition, and diagnosis processes. Specifically, MOF probes with nanosized cavities can meet the intrinsic theranostic properties of specific organic ligands with the biocompatibility and/or low cytotoxicity of the metal. Up to now, they have a unique impact in prospective of drug encapsulation and delivery. 

As will be discussed, the tuneable and tailoring absorption and emission properties of the zinc-based CPs make them ideal candidates as both colorimetric and fluorophore smart materials. In the next section the unique role of the zinc (II) cation in the backdrop of the stimuli-responsive CPs will be underlined and discussed.

### 1.4. The Charming Peculiarity of Zinc Cation

The self-assembly of polydentate ligands and transition metal cations provides endless architectures and infinite behaviours. The construction of CPs with tailored properties and targeted applications is a seething research area. The interest is driven by the impact on the technology and the economy. Technicians and users are addressing the demand to the challenge of sustainability. Alternative cheap “green matter” is gaining traction over toxic and expensive products. The use of zinc cation in building of supramolecular CPs moves in this direction. 

**Scheme 1:**
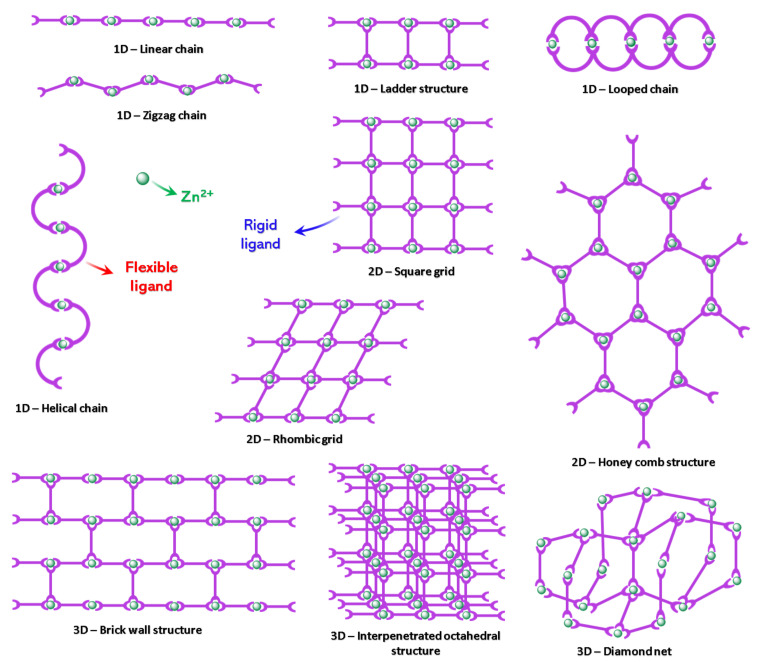
Relevant topological types of Zn-CPs.

From a structural point of view, the d^10^ closed-shell metal ion Zn^2+^ is suited for the construction of varied coordination architectures [102]. Zinc (II) cation can adopt three coordination numbers, i.e., 4, 5, and 6. The energetic cost for hydrated ion to switch among the three coordination numbers is within 0.4 kcal mol^−1^ in the gaseous phase. Such ability contributes to the effectiveness of Zn (II) as a catalytic centre easily undergoing coordination–decoordination equilibria depending on the environmental conditions [103]. Due to its flexible coordination environment and geometries, zinc ion can accommodate different structural arrangements such as linear or zigzag chains, ladder, square, rhombic, brick-wall structures, and diamond networks (Scheme 1) [22].

Starting from the mono and bi-dimensional macro-sized CPs employed in sensing of metals, small organic molecules, and environmental pollutants to the highly engineered three-dimensional nano-architectures able to cell permeation, zinc (II) cation can realise limitless combinations of 1D, 2D and 3D polymeric structures [55,56,57,58]. Zinc-based MOFs and nanoprobes are now a significant group of versatile engineered tools for medical diagnostic. They are currently employed for the detection of bioanalytes, as drug carriers and as chemotherapeutics, and to monitor and/or mark specific cells and subcellular compartments and parameters. Both as macro and nano-sized tools, chromic zinc-based CPs can be rightly placed among the emerging fluorophores used as smart probes. 

Luminescence in CPs (LCPs) arises from the characteristic of the organic ligands which are coordinated to the metal centres or clusters [43,104]. Guest molecules get entangled into the frameworks leading to the emission response (enhancement or quenching of luminescence). From a theoretical point of view, the origin of the luminescence processes in the hybrid polymers arises from one or several of the following electron transfer mechanisms: ligand cantered (LC) luminescence; metal cantered (MC) luminescence; luminescence arising from charge transfer (CT) states, which includes ligand-to-ligand (LLCT), ligand-to-metal (LMCT), and metal-to-ligand (MLCT) charge transfers. 

When the metal cation is a heavy atom, it drives the ligand luminescence from the short-lived fluorescence of the singlet state to the phosphorescence arising from triplet states. It can also force the system into triplet states with non-radiative deactivation. MC luminescence is found predominant in CPs based on lanthanide or actinide cations and can occur in transition and p-block metals. In CPs based on transition metal cations, luminescence typically arises from CT states [101]. LC emission is due to the organic ligands used in the assembly of the CPs. In principle, LC transitions could not differ significantly in the bonded and in the unbonded ligand. Metal cations able to not disturb and/or increase the LC; luminescence processes are the closed shell electron configurations, such as alkaline and alkaline earth ions and d^0^ and d^10^ transition metal cations. Compared to the metal-based processes, LC transfers play a relevant role in the building of luminescent CPs with high tunability and customization. 

The closed-shell zinc (II) ion undergoes the deactivation of d-d transitions, so that the emission in Zn-CPs (short for zinc-based CPs) is mostly due to π–π* LC transitions and/or to LLC transitions (“optically innocent” role [105]). In many cases, zinc cation acts as a constraint (“clip” approach [105]) for the ligands locking them into a favourable emissive conformation and adding scarce electronic effects [84,106,107,108,109,110,111,112,113]. The chelation enhanced fluorescence (CHEF) mechanism upon coordination is often the result of the stabilisation of the excited state in poorly emissive ligands [114,115,116,117,118,119]. Therefore, the zinc (II) cation can be the optimal building block for tailoring of luminescent zinc-based CPs (Zn-LCPs). In particular, the role of Zn^2+^ in producing strong fluorescence due to the aggregation of the organic ligands is widely recognised [84,120]. The aggregation-induced emission (AIE) effect [114,121], mostly imputed to the restriction of intramolecular rotations (RIR), Refs. [122,123,124] are well known in zinc-based coordination compounds and results in highly emissive solid materials.

On the other hand, the ability of Zn^2+^ to undergo structural reorganization and coordination–decoordination equilibria in presence of different ligands, solvents, competitive cations, and analytes makes Zn-CPs good candidates for several kind of chromic response, both in absorbance and in emission [105].

Among the wide variety of studies encompassing the topic of CPs, in this review, we will focus our attention on the role of zinc (II) cation in building stimuli-responsive supramolecular polymeric architectures. We will refer to the recent (2016–2021) Zn-CPs underling synthetic novelty, research completeness, chromic response, theoretical studies, and significant applications. Scientific literature including articles and reviews was collected and consulted, and the related discussions were organised according with the target analyte and the response mode, in the following sections:

(1) Zn-CPs for metal cations, oxyanions, small and even harmful molecules;
(2) Zn-CPs responsive to pH and temperature;
(3) Responsive Zn-CPs hydrogels;
(4) Zn-CPs for detection of biological targets;
(5) Multifunctional Zn-CPs for bioimaging.

**Figure 1:**
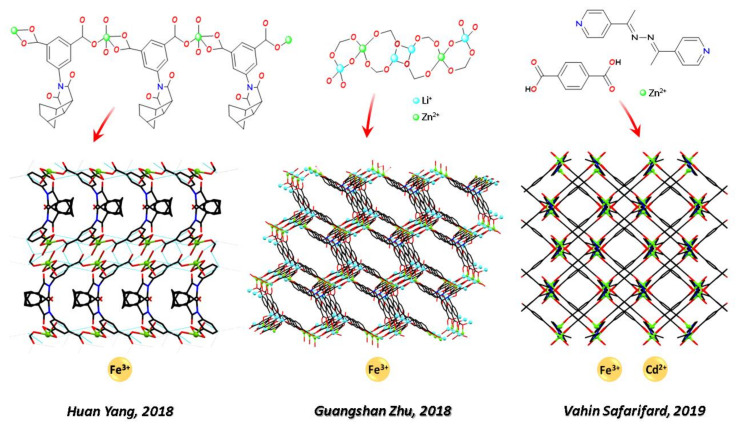
Some representative structures of Zn-CPs for metal cation sensing. Figures readapted by using Mercury software [125].

**Figure 2:**
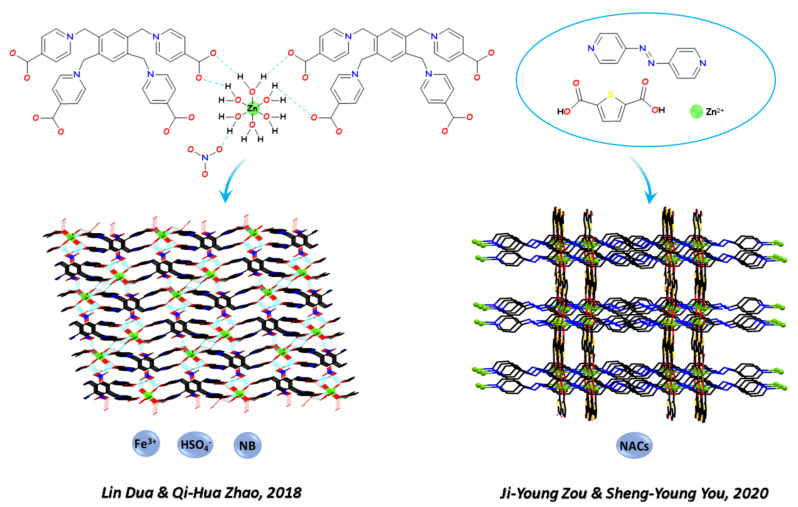
Some representative structures of Zn-CPs for multi-sensing of metal cations, oxyanions, and NACs metal cation sensing. Figures readapted by using Mercury software [125].

**Figure 3:**
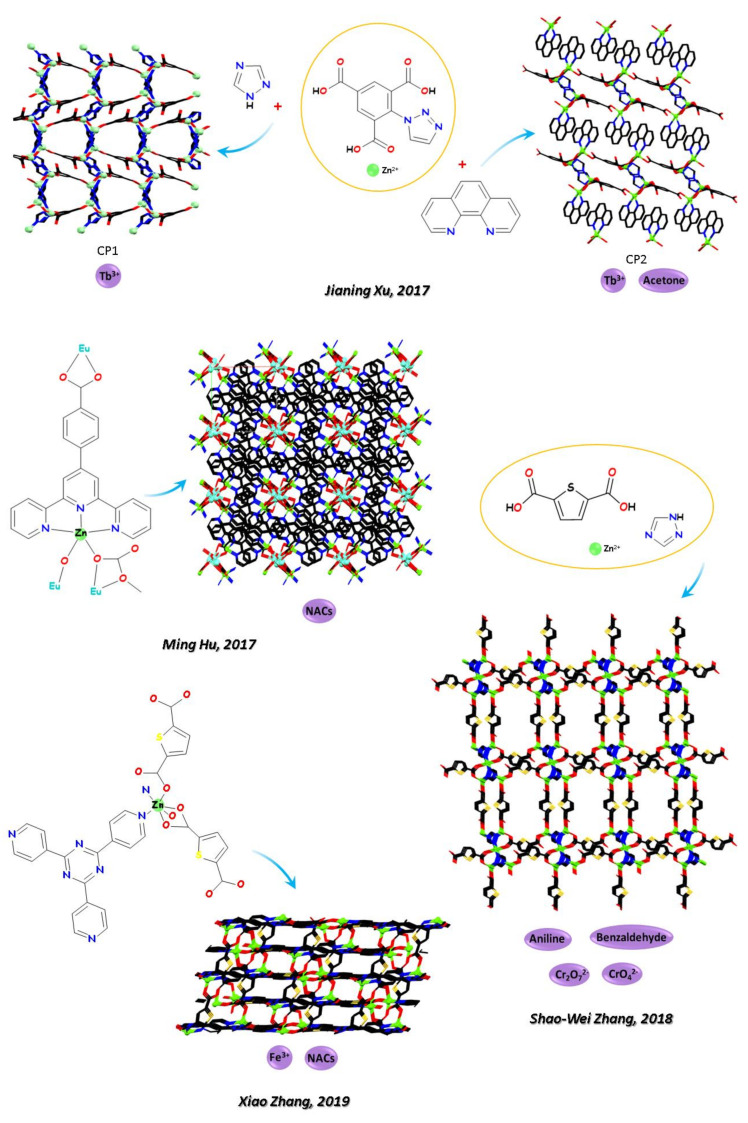
Other representative structures of Zn-CPs for multi-sensing of metal cations, oxyanions, and NACs metal cation sensing. Figures readapted by using Mercury software [125].

**Figure 4:**
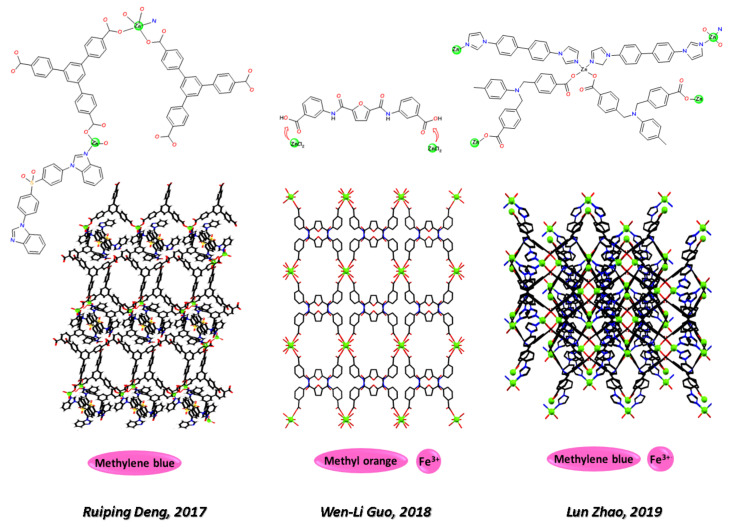
Some representative structures of Zn-CPs for multi-sensing of biologically harmful small organic molecules. Figures readapted by using Mercury software [125].

**Figure 5:**
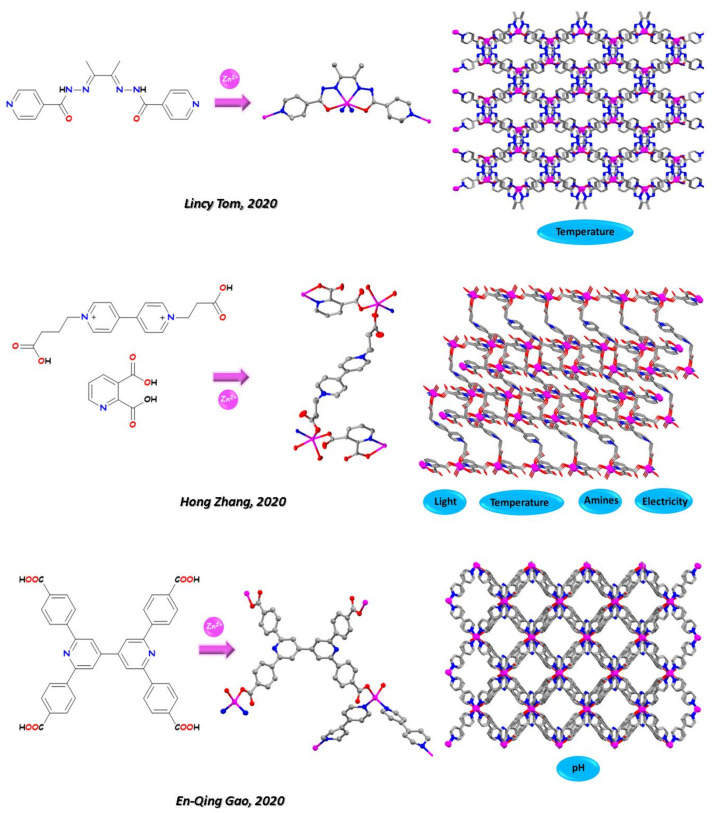
Some representative structures of Zn-CPs for pH and temperature sensing. Figures readapted by using Mercury software [125].

**Figure 6:**
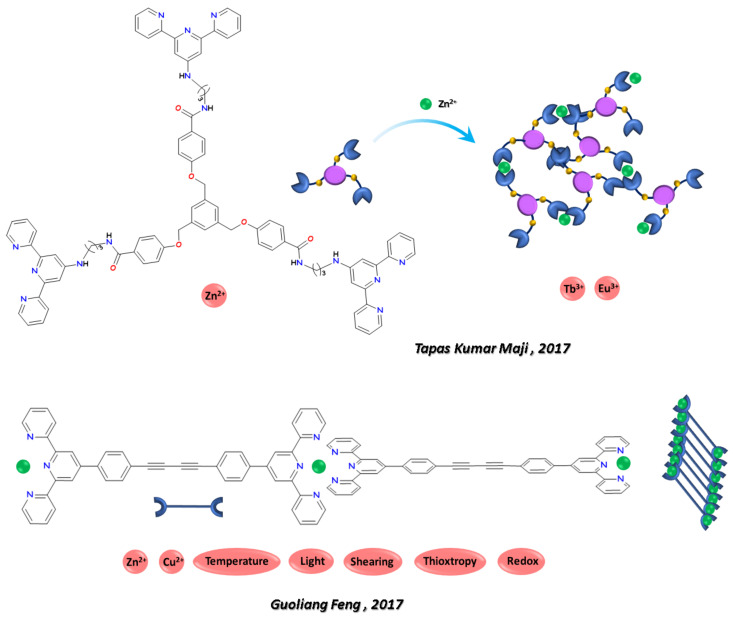
Some representative structures of Zn-CPs stimuli-responsive gels. Figures readapted by using Mercury software [125].

**Figure 7:**
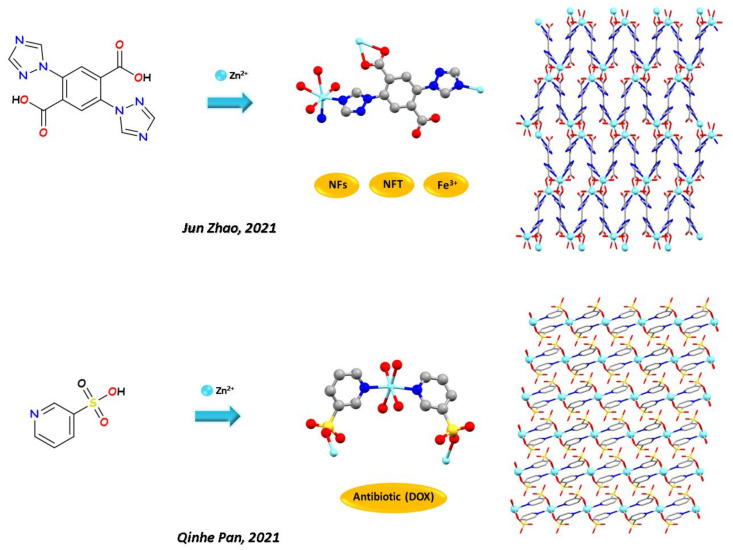
Some representative structures of Zn-CPs for sensing and delivery of bio-relevant species. Figures readapted by using Mercury software [125].

Our goal is to provide an overview of the chromic Zn-CPs as stimuli-responsive materials by cutting across the prolific and complex CPs research field. For this purpose, we will add as examples selected structures in the Figures 1–7, indicating the specific responsiveness in a quick glance-mode. The structures reported in all the figures have been readapted using Mercury software [125].

## 2. Zn-CPs Responsive toward Metal Cations, Oxyanions, and Small Molecules

The detection principle in sensing by CPs is the interaction of the probe with molecules/ions due to potential interacting sites present at the ligand or the metal. The interaction can alter the structural pattern and/or the orbital energy levels of the material. Specifically, in chromogenic LCPs, the emission position and/or intensity can be altered by the presence of an analyte able to change the ground- and/or the excited-state of the sensing probe [43,104]. In this section, particular attention has been paid to the luminescence properties of Zn-LCPs and their applications as chemical sensors.

Zn-CPs offer a suitable platform for a novel generation of colorimetric and/or luminescent smart sensors. The role of the zinc (II) ion is crucial in the turn-on/turn-off chromic response of the sensing material. Thanks to the varied structural ability and to the coordination–decoordination equilibria of the zinc (II) coordination core, limitless polymeric architectures can be dissolved and reformulated [126,127]. The interactions between ligands and zinc nodes can be destroyed and recovered by adding competitive analytes, resulting in a change of the natural colour or in a fluorescence quenching or enhancement, due to structural and/or electronic rearrangements. In some cases, also the dimensionality of the CP matrix can undergo a substantial change, turning 1D and/or 2D polymers into 3D networks. Finally, the sought-after selectivity and real-time response properties are often guaranteed by Zn-CPs probes. 

In the last five years, Zn-CPs were successfully employed in the sensing of metals, small organic molecules, and ions [128,129,130,131,132]. The sensing ability toward metals is a relevant property. Metals are essential to chemical, enzymatic, and biological processes; on the other hand, they may represent environmental pollutants. Other hazardous pollutants are small molecules/ions such as chromates and dichromates, azo compounds, nitroaromatic compounds, and volatile organic compounds (VOCs). Trapping and detection of such chemicals is a hot topic in the field of stimuli-responsive probes. In this section, some relevant articles will be summarized highlighting and discussing the relationship between structure (derived by X-ray diffraction analysis), dimensionality, and sensing ability.

Due to their unique crystalline structure and voids, high surface area, and functionalities, regular 3D CPs (mainly MOFs) are the ideal versatile candidates for selective sensing [133,134,135,136]. In designing a responsive luminescent MOFs (LMOFs), the electronic structure of the host frames and the host–guest interactions must be considered. The geometry of the voids and the interacting binding site lead to selective sensing properties in a single or multi-functional mode. Obviously, the nature of the metal ion affects the structural pattern, the chemo- and physical properties, and finally the sensing specificity and mechanism. Both a fluorescence enhancement or quenching can be the result of the interaction between the MOF cavities and the analyte, due to the molecular and electronic changes of the guest–host system. If the target analyte interferes with the ligand’s electronic structure reducing the energy transfer from the ligand to the metal, the sensing process results in a decrease of the fluorescence intensity. But if the analyte moves the activation of a novel emissive channel, a fluorescence enhancement can be obtained. Zn-LMOFs typically emerge as strong luminescent probes due to the CHEF effect imposed by zinc (II) ion. They can both retain or quench the fluorescence response of the linkers mainly related to LCT and/or LLCT transitions. 

### 2.1. Mono or Multifunctional Metal Cations Sensing 

Recent relevant examples of Zn-CPs metal sensing were presented in 2017–2019. In 2017 Jian-Ping Lang and coworkers [128] presented a deeply characterised series of a 1D and four 3D Zn-CPs obtained by ligands 1,4- bis(2-(pyridin-4-yl)vinyl)naphthalene and 1,3,5-benzene-tricarboxylic acid in different molar ratio. The structural pattern of the five CPs was explored, and their luminescent response toward Hg^2+^ was examined. Specifically, the more representative example resulted highly selective and sensitive for the detection of Hg^2+^ ion. A finely ground sample suspended in N, N dimethylformamide (DMF) solvent undergoes a fluorescence quenching and a change of fluorescence colour from blue to yellow naked eye perceivable under UV light. As an essential trace element in the human body, crucial in several biological processes, iron ion is a desirable metal target. In 2020, Zhong-Feng Shi and coworkers [137] published another prominent article on two Zn-CPs produced by hydrothermal reactions of ligands 5-sulfosalicylic acid and 1,4-bis(1H-imidazol-1-yl) benzene (1,4-bib) for different reaction time. After two days reacting, a 2D structure was obtained, while a 3D coordination polymer was obtained after the same mixture reacted for three days, each structural unit being linked by ten 1,4-bib ligands to form a 3D MOF. The 2D CP shows excellent fluorescence quenching response toward Fe^3+^ and the 3D CP toward Hg^2+^. The quenching mechanism was explored in both cases and ascribed to the absorption of the emitted light for Fe^3+^ and to the electron transfer and weak interaction of Hg^2+^. In 2018, two Fe (III) quenching responsive and highly selective Zn-MOFs were described. Specifically, Huan Yang and coworkers [138] (see Figure 1) produced a luminescent Tb^3+^ and Eu^3+^ encapsulated Zn-MOF based on 5-(5-norbonene-2,3-dicarboximide)isophthalic acid, and Guangshan Zhu and coworkers (see Figure 1) [139] obtained a mixed Li^+^ and Zn^2+^ based MOF from the quadridentate carboxylate ligand 5-(bis(4-carboxybenzyl)amino)isophthalic acid. 

The simultaneous detection of selectively trapped metal cation is a desirable property of some LMOFs. Many Zn-MOFs for simultaneous detection of iron and another metal cations are known, such as the Eu^3+^ encapsulated Zn-LMOF based on tetrakis(4-pyridyloxymethylene)methane and 2,6-naphthalenedicarboxylic acid able to detect Fe^3+^ and Cr^4+^ ions (Xin Liu and coworkers [140], 2017); the Zn-MOF responsive to Fe^3+^ and Al^3+^ ions (Xia Li and coworkers [141], 2018) assembled from 3,3′-diphenyldicarboxylate and 1,4-bis(1,2,4-triazol-1-yl)butane; the Zn-MOF named TMU-16 from a bipyridyl ligand with a bridging azine group (Vahid Safarifard and coworkers, 2019, see Figure 1, [142],) displaying a luminescence quenching response to Fe^3+^ and a luminescence enhancement response to Cd^2+^ ions.

### 2.2. Multi-Sensing of Metal Cations, Oxyanions, and Nitro Aromatic Compounds

Among the innumerable LCPs, several Zn-LCPs have drawn attention as highly selective multi-analytes probes. Simultaneous operativity is a sought-after property, and it enables the probe to be employed in an actual technological use. Multi-responsive Zn-CPs explored in the last years can detect simultaneously metal cations (mainly Fe^3+^), anions, and small molecules. Specifically, common carcinogenic pollutants of aqueous solutions used in several industrial applications [143,144,145] such as NACs (nitro aromatic compounds) and chromium (VI) oxoanions (Cr_2_O_7_^2−^, CrO_4_^2−^) are the target analytes of 3D Zn-CPs.

Recent examples are the work of Bao-Long Li and coworkers, which in 2018 [146] studied the crystalline pattern of five Zn-CPs based on the ligand 1,3-bis(1,2,4-triazol-4-ylmethyl)benzene with different co-ligands (btec = 1,2,4,5-benzenetetracarboxylate, Meip = 5-methylisophthalate, nip = 5- nitroisophthalate, hip = 5-hydroxyisophthalate, nbdc = 4-nitro-1,2-benzenedicarboxylate). The CPs display different structural patterns and dimensionality from 1D to 3D. The 3D CP shows a (4,4)-connected self-catenated 3D network of the 4,4T32 topological type and is a highly sensitive and selective luminescence sensor for Fe^3+^, Cr_2_O_7_^2−^, and CrO_4_^2−^ in aqueous solution, with detection limits of 6.28, 3.05, and 5.72 μM, respectively, and an assumed quenching mechanism. In the same year, Lin Dua, Qi-Hua Zhao, and coworkers (see Figure 2) [147] synthetised three novel LCPs from the flexible zwitterionic ligands 1,1′,1″-(2,4,6-trimethylbenzene-1,3,5-triyl)-tris(methylene)tris-(4-carboxypyridinium)tribromide and 1,2,4,5-tetrakis-(4-carboxylatopyridinium-1-methylene)benzene and zinc (II) or cadmium (II) d^10^ cations. They compared the different structural pattern and dimensionality promoted by the metal and the related sensing ability toward Fe^3+^, nitrobenzene (NB), and HSO_4_^−^ anions.

In 2019, Hao Lei and coworkers [148] obtained a series of 2D Zn-CPs by ionothermal reaction a series of 2D Zn-CPs named {Zn2 × 2(BDC)(L)}n where X = Cl (1 and 3) or Br (2 and 4), BDC = 1,4-benzenedicarboxylate, L = 4,4′-bipyridine (4,4′-bpy, 1 and 2) or 1,4-diazabicyclo [2.2.2]octane (dabco, 3 and 4). The polymer 1 (with chloride and 4,4′-bpy) displays strong luminescence in the solid state and selective luminescence quenching for Fe^3+^ ions and nitroaromatic compounds (NACs) in ethanol solutions, while the polymer 4 (with bromide and dabco) can selectively adsorb Congo red dye from other dye molecules. In 2020, Yujuan Zhang and coworkers [149] used copper (II) in a case and zinc (II) in another case as metal nodes to produce two CPs, namely [(Cu(bib)2)⋅2NO_3_⋅H_2_O]n and [[Zn_3_(bib)2.5(C_2_O_4_)3(H_2_O)]] DMF⋅H2O]n. By structural analysis, the polymeric arrangements were compared and both CPs were found to be 2D frameworks further expanded into 3D supramolecular structures through C–H⋯O hydrogen bonding. The sensing pathway was examined. The CPs were found to be quenching responsive to Fe^3+^ and NACs, through a PET (photoinduced electron transfer) and RET (resonance energy transfer) mechanism for NACs, and a RET mechanism for Fe^3+^, respectively. Very recently, Baokuan Chen and coworkers [150] explored the influence of carboxylic acid substituents on the structural and emissive pattern of three Zn-CPs built from isophthalic acid (1), 5-hydroxyisophthalic acid (2), and 5-nitroisophthalic acid (3), respectively, and the N-donor ligand N,N’-bis(4-methylenepyridin-4-yl)-1,4-naphthalene dicarboxamide. The CPs have similar 4,4-connected 2D structures exhibiting different structural details and show multi-functional fluorescence response towards metal ion (Fe^3+^), anions (MnO_4_^−^, Cr_2_O_7_^2−^, CrO_4_^2−^), and 2,6-dichloro-4-nitroaniline. The luminescence quenching effect was not ascribed to any skeleton collapse but to an energy competitive absorption mechanism, which leads to a RET effect.

Zn-CPs can be useful tools also for detection of rare heart metals. Jianing Xu and coworkers (see Figure 3) [151] in 2017 obtained two Zn-LCPs based on ligand 1- (triazol-1-yl)-2,4,6-benzene tricarboxylic acid and 1,2,4-triazole (1) and 1,10-phenanthroline (2), respectively. CP1 shows a 3D framework helical chain and CP2 shows a 2D layered network extending to 3D structure through π–π stacking interactions. Both CPs perform as hosts for the encapsulation of Ln^3+^ ions and serve as antennae to sensitize Tb^3+^ ions. In addition, CP2 exhibits highly luminescent sensing properties for acetone.

Recently, examples of 3D tools as Zn-LMOFs selectively detecting nitro derivatives were studied in depth both from the structural and functional point of view. In 2020, Ji-Young Zou, Sheng-Young You [152], and coworkers (see Figure 2); in 2017, Ming Hu and coworkers (see Figure 3) [153]; and in 2019, Xiangyang Qin and coworkers [154] ascertained the quenching mechanism imposed by the presence of NACs in a series of Zn-LMOFs where a ruling FRET process affected the luminescence response of the probe. In other cases, multifunctional Zn-LMOFs was able to selectively detect NACs and/or Cr_2_O_7_^2−^ and CrO_4_^2−^ anions, and/or Fe^3+^, and a series of selected examples are briefly reported below. As relevant examples, in the same year (2018), Suna Wang and coworkers [155] produced two Zn-LMOFs based on 2,2′-[benzene-1,3-diylbis(methanediylsulfanediyl)]dibenzoic acid, 1,3-bis(4-pyridyl)propane, 1,2-Bis(4-pyridyl)ethylene, and DMF (N,N-Dimethylformamide), and He-Gen Zheng and coworkers [156] produced three Zn-LMOFs, based on E,E-2,5-dihexyloxy-1,4-bis-(2-pyridin-vinyl)-benzene, 1,4-cyclohexanedicarboxylic acid, 4,4′-oxybisbenzoic acid, and 4,4′-sulfonyldibenzoic acid for selective detection of Fe^3+^ and Cr_2_O_7_^2-^. The same couple of analytes were selectively detected by the MOF named [Zn(dptz)(BDC)(H_2_O)]n [1, dptz = 3,6-di(1H-pyrazol-4-yl)-1,2,4,5-tetrazine, H_2_BDC = terephthalic acid] (Xian-He Bu and coworkers [157], 2020) and by the MOFs named [Zn_2_(HL) (phen)]n and [Zn_2_(HL)(2,2-bipy)]n obtained from the V-pattern multi-carboxylic acid ligand H_5_L = 3,5-di(2′,5′-dicarboxylphenyl)benzoic acid (Tuoping Hu and coworkers 2019 [158]). 

NACs and Fe^3+^ can be selectively detected by a Zn MOF with a 3-fold interpenetrating 3D framework based on tpt = 2,4,6-tri(pyridin-4-yl)-1,3,5-triazine and H_2_tda = 2,5-thiophene dicarboxylic acid (Xiao Zhang and co-workers in 2019, see Figure 3 [159]) and by the MOFs obtained by 4,40,400-nitrilotribenzoic acid (H_3_NTB) and 1,10-phenanthroline (phen) (Lirong Yang and coworkers in 2021 [160]). In all cases, a quenching response was detected for both analytes. Multi-responsive probes for NACs, Fe^3+^, and Cr (VI) oxo-anions were designed by Zhong-Feng Shi and coworkers in 2020 [161] based on 3-nitro-4,4′-biphenyl dicarboxylic acid and 11,4-bis(imidazole-1-ylmethyl)-benzene. Aniline, benzaldehyde in DMF and Cr_2_O_7_^2−^/CrO_4_^2−^ anions in water can be selectively detected by the Zn-MOF based on a pentametallic clusters and thiophene-2,5-dicarboxylic acid (Shao-Wei Zhang and coworkers (see Figure 3) [162], 2018).

### 2.3. Multi-Sensing of Biologically Harmful Small Organic Molecules

Many 3D Zn-CPs have been designed to be highly selective toward specific small molecules. Zn- MOFs responsive towards environmental organic pollutants, such as VOCs and water-soluble organic dyes and pesticides, are highly required in water quality tests. In many cases, they are selective multifunctional probes. Very recently, a variety of Zn-LMOFs probes selectively responsive towards biologically harmful molecules generated as wastewater from industrial processes [163] have been produced.

Huai-Ming Hu in 2016 [164] prepared three Zn-CPs based on 40-(4-carboxyphenyl)-60-carboxycalte-2,20-bipyridine and glutaric acid. Depending on different pH values and auxiliary ligands, different 2D (hcb topological net and layer structure) or 3D (eight-membered rings self-penetrating MOF structure) CPs were obtained. The Zn-LMOF shows high-sensitivity sensing to metal cations (Fe^3+^ and Cu^2+^) and harmful small organic molecules (as methanol and nitrobenzene). More recently, in 2020, Jianrong Li [165] and coworkers synthetised a 3D Zn-LCP by the reaction of Zn(NO_3_)_2_, N,N′- bis (3-pyridinecarboxamide)-1,4-butane and 4, 4′- oxidiphthalic acid. Besides the in-deep structural analysis, the polymer demonstrated remarkable fluorescent properties and chemical stability under an acidic or alkaline environment. It was used as a multifunctional chemosensor for detection of metal cations (Fe^3+^, Bi^3+^), oxyanions (MnO_4_^−^, Cr_2_O_7_^2−^), and toxic organic solvents such as NB, acetaldehyde, and acetylacetone. The mechanism of sensing process indicated that the synergistic effect of electron transfer by both luminescent ligands and the FRET mechanism leads to fluorescence quenching. 

In some cases, Zn-LMOFs are designed to be highly selective toward highly specific small molecules. In 2018, Jian-Zhong Cui and coworkers [166] produced an anionic Zn-MOF from furan-2,5-dicarboxylic acid and 1H-benzotriazole working as a turn-off luminescent sensor toward a volatile and flammable analytical reagent, acetylacetone. The selective structural cavities of MOFs make them ideal candidates for detecting VOCs upon adsorption. Patima Nizamidin and coworkers in 2019 [167] proposed a series of Zn MOFs, derived from terephthalic acid and N,N’-di(4-pyridyl)-1,4,5,8-naphthalenediimide. Formed in thin glassy films, the MOFs membranes display a selective adsorption response to meta-xylene gas. In 2019, Bing-Hui Wanga and Bing Yan [168] designed a Zn-MOF based on a fluorescein dianion functionalized dye for the detection of the small trichloroacetic acid (TCA), a carcinogen metabolite in human urine. 

Zn-MOFs responsive towards environmental organic pollutants, such as organic dyes and pesticides, are sensing tools in water quality tests. In 2017, Ruiping Deng and coworkers (see Figure 4) [169] reported a Zn-MOF built from bis(4-benzylimidazol-ylphenyl)sulfone and 4,4′,4′’-benzene-1,3,5-triyltribenzoic acid responsive to methylene blue. Wen-Li Guo and coworkers (see Figure 4) in 2018 [170] and Lun Zhao and coworkers (see Figure 4) in 2019 [171] studied two multifunctional systems for sensing of iron and a dye. Based on an 8-fold interpenetrating diamond network of N,N0-bis(4-carbozylbenzyl)-4-aminotoluene) ligand [171] and on one of bis-(3-carboxy-phenyl)furan-2,5-dicarboxamide [170], respectively, the first one selectively traps methylene blue and the second one methyl orange dye.

In 2020, three relevant articles were produced on multifunctional MOFs probes for environmental pollutants. Jarugu Narasimha Moorthy and coworkers [172] synthetised a Zn-MOF with ca. 27% solvent-accessible void volume based on a tetracarboxylic acid ligand with a twisted dibenzo[g,p]chrysene core (2,7,10,15-tetrakis [2,6-dimethyl-4-(α-carboxy)methoxyphenyl]-dibenzo[g,p]chrysene) highly specific in water toward hazardous “quat” dicationic herbicide with diquaternary bipyridyl motifs. Xiutang Zhang and coworkers [173] used a 3D self-penetrated framework based on 1,3-bis (imidazol-1-ylmethyl)benzene and *π*-conjugated aromatic *p*-terphenyl-2,2″,5″,5‴-tetracarboxylate acid for DCN (2,6- dichloro-4-nitroaniline) pesticide and for nitrofuran NFT (nitrofurantoin) and NTZ (nitazoxanide) antibiotics. Finally, Qinhe Pan and coworkers [174] designed a Zn MOF for the detection of U(VI) as a potential environmental pollutant. 

## 3. Multi-Mode Zn-CPs Responsive to pH and Temperature

Temperature and pH and are crucial in most chemistry reactions and in biological processes. As fundamental life parameters, their accurate measurement is crucial in scientific and technological contexts. The development of effective sensors for selective and sensitive detection of such target analytes is of great importance in the field of smart materials and diagnostic tools. The dual-mode detection strategy has attracted considerable attention as more effective than the single-mode method. Specifically, dual-mode colorimetric/fluorescent sensing systems have been developed by scientists, and optical pH sensors with integrated temperature sensors are among the commercially available devices [175,176,177,178,179,180,181,182,183].

CPs based tools are emerging smart materials for pH and temperature monitoring, even in addition to other chromic responses. Many CPs probes for pH function in an on–off or in a gradual and/or ratiometric mode [184]. Colorimetric probes can provide a sharp change or a gradual change of colour around a specific pH value. Absorbance colorimetric phenomena typically occur because of desolvation or change of the coordination environment around the metal ions. The design of highly sensitive fast responsive naked eye tools is the current challenge for on-site sensing. Several thermochromic CPs are known to exhibit reversible colour-switching phenomena triggered by thermal stimulation. On the other hand, a simultaneous luminescence response is crucial for biological applications such as fluorescence microscopy at the molecular level, even in living samples. Ratiometric PL sensors are two emission wavelengths responsive probes with the advantage of high measurement accuracy [184]. For diagnostic purposes (sensors acting around physiological pH), sensors exhibiting a dual-mode gradual colorimetric response and PL ratiometric response are the most suitable tools [185,186,187].

Many nanosized CPs for bioimaging and living cell determination have been recently reported in excellent articles and reviews [188,189,190,191,192,193,194]. LMOFs were often used as markers for biomolecules and in living cells for subcellular detection and therapeutic applications. Here, we will report the recent examples of Zn-CPs exhibiting pH and thermo triggered mono and dual-mode chromic responses referring to well-characterised non-applicative systems. The biomedical and theranostic uses will be discussed in Section 5.

Among the group of thermochromic CPs, Jun-Ling Song [195] studied a multiple stimuli-responsive 2D CP based on 5-(4-sulfophenylazo)salicylic acid with an elastic layer-structured framework, forming a 3D interlinked network by extensive π–π stacking interactions. Thermal removal of water molecules leads to a reversible naked eye thermochromism and to selective gas absorption behaviour and a breathing effect. The reversible thermochromism was mainly ascribed to the change of the coordination environment of Zn^2+^ ions in the dehydration/hydration process. A year later, an intriguing study was developed by Hong Zhang and coworkers (see Figure 5) [72] on a viologen-based multi-stimuli colorimetric Zn-CP, exhibiting a 1D channel structure. The electron-deficient viologen fragments (containing V^2+^, N,N′-disubstituted 4,4′-bipyridiniums) introduce in the metal framework a broad-range chromogenic unit. The probe resulted responsive to light (photochromism under 300 W xenon lamp), heat (thermochromism from colourless to light yellow when heated at 106 °C), amines (chemochromism when exposed to different simple amines), and electricity (electrochromism). The heated state promoted a photochromic response due to the loss of water molecules, while the PL quenching was ascribed to the collapse of the excited state by the formation of a radical. The same year, Lincy Tom and coworkers (see Figure 5) [196] published a dual-mode scheme thermotropic luminescent Zn-CP, built from 2,3-butanedionebisisonicotinylhydrazone. The thermochromic colorimetric (from yellow to red by heating) and PL (quenching by heating) response in the range 40–90 °C can be reversibly switched in the solid state, with a linear wavelength/intensity relationship. X-ray crystal analysis revealed a 2D layer-structured framework with trigonal prismatic Zn (II) centres. The reversible thermochromism was mainly ascribed to the change in the coordination environment of Zn^2+^ ions in the hydration/dehydration process.

Zn-LMOFs are good candidates for the thermochromic response. In many cases, their PL response in driven by the right choice of both organic frameworks and by the embedding of apt receptors into the MOF scaffold. A Zn-LMOF for a ratiometric temperature measurement was obtained by Guodong Qian and coworkers in 2017 [197] from a tetracarboxylic acid ligand containing a benzothiadiazole moiety (Zn-MOF ZJU-21). Once the luminescent dye 4-[p-(dimethylamino)styryl]-1-methylpyridinium (DMASM) is absorbed, the resulting composite can be used as a ratiometric probe for temperature measurement. In 2021, Banglin Chen and coworkers [198] synthesized two ratiometric thermometries from NbO-type Zn-LMOFs (named Eu^3+^@ZnPZDDI and Eu^3+^@ZJU-56) using 5,5′-(pyrazine-2,5-diyl)diisophthalic acid and 5,5′-(pyridine-2,5-diyl)diisophthalic acid and absorbing pyridinium hemicyaninedye 4-[p-(dimethylamino)styryl]-1-methylpyridinium (DSM) and Eu^3+^ in the crystalline architectures.

Zn-LMOFs are also suited probes for a chromic pH response. In 2020, En-Qing Gao and coworkers (see Figure 5) [137] produced a Zn-LMOF from a bipyridyl-tetracarboxylic ligand (2,2′,6,6′-Tetra(4-carboxyphenyl)-4,4′-bipyridine (H4tcpbp)) composed of 4-fold interpenetrated diamond frameworks where the free donor N sites make the probe pH-responsive in a reversible on–off mode in the pH range of 5.4−6.2. A thermal and pH dual stimulus responsive Zn-LMOF was obtained by Yulin Yang and coworkers in 2018 [199] from a semirigid 5-(4′-carboxyphenoxy)nicotinic acid. The probe proved to be a biocompatible drug delivery carrier of 5-FU drug molecules under pH and temperature controllable releasing conditions.

## 4. Stimuli-Responsive Zn-CP Gels

Gels are classified into chemical gels (with covalent cross-linking) and physical gels (with noncovalent cross-linking). Supramolecular gels constructed from the self-assemblies of small molecules called low-molecular-weight gelators (LMWGs) are a type of physical gel. Polymer gels are the most versatile soft materials with commercial applications such as contact lenses, gel mattresses, and solid air fresheners [200]. Recently, supramolecular polymer gels obtained as networks of macromolecules cross-linked via noncovalent bonds have been drawing attention from both basic and applied viewpoints. 

Coordination Polymer Gels (CPGs) [200] are multi-dimensional networks constituted by a solid-like metal ion and bridging organic frameworks with a trapped solvent because of non-covalent interactions. A gel containing metal ions and bridging ligands can be classified as a metallogel. CPGs are an interesting subset in this category. The ability of CPs to undergo reversible assembly/disassembly equilibria is stressed in the gel phase and can impart CPGs added functionalities. Additional energy (in form of heat, sonication, and shaking) gives a solution of solvated gelators which can migrate through the gel system and interact with metals, ligands, and solvent (sol–gel equilibrium). Studies carried out to understand the mechanism of gelation and to deep the stimuli-responsive behaviour of CPGs were published in relevant articles [71,201,202]. As almost all polymeric gels, CPGs show both a liquid-like flow and an elastic behaviour. Moreover, they show the self-healing function of gelling materials [201,202]. Self-healing materials can heal and restore the material to its original set of properties when damaged/stressed by thermal, mechanical, ballistic, or other kind of stimulus. Therefore, sensing is the most valued potential use of these dynamic systems, highly tunable and tailorable, often thermo-responsive. As a proof-of concept, CPGs pave a new way for the next-generation of soft matter sensing devices self-powered and conformable to human skin or tissue.

Zinc based CPGs (Zn-CPGs) are a class of the novel supramolecular polymeric gels. Often luminescent in the gel phase, Zn-CPGs are usefully stimuli-responsive, still benefiting of the above discussed structural, coordinative, and emissive ability of the zinc (II) cation, in addition to their “green soul”.

Pyridine-based ligands are often suitable candidates as organic building blocks, due to the coordination-decoordination ability toward zinc (II) cation. Bin Hua and coworkers [203] in 2018 used a 2,2′-bipyridine-bridged pillar[5]arene dimer for constructing linear supramolecular polymers through guest–host interaction and employed Zn^2+^ ions to cross-link the linear supramolecular polymer into a supramolecular network gel. The CPG exhibits thermoreversible gel–sol transformation and results also responsive toward the addition of competitive guest adiponitrile, which destroys its gelation. Tapas Kumar Maji and coworker (see Figure 6) [204] in 2017 used the fluorescence turn-on response of a gelator for ppm detection of Zn (II) ion in aqueous solution. The gelator consists of 4,4′,4-[1,3,5-phenyl-tri(methoxy)]-tris-benzene and 2,2′:6′,2″-terpyridyl frameworks and was employed with different metal ions to form CPGs. The zinc hydrogel exhibits sheet-like morphology and CT emission. With Tb (III) and Eu (III), the same result in green- and red-emissive CPGs with nanotubular morphology.

In 2017 Guoliang Feng and coworkers (see Figure 6) [205] reported the preparation of supramolecular CP hydrogels based on a ditopic terpyridine-acetylene ligand (DTA) with zinc (II) or copper (II) cations. The metal ion directs the self-assembly of DTA with the formation of CPs entrapping water to form hydrogels. The metal cations control gelation and stimuli-responsive properties of the CPG. Specifically, DTA ligand proved to selectively recognize Zn^2+^ ions gelating into fluorescent metallogels and Cu^2+^ gelating into nonfluorescent, electrochemical, and chiral hydrogel multi-responsive to stimuli such as heat, light, shearing, thioxtropy and redox. Other terpyridine-based systems were exploited by Yi-Tsu Chan and coworkers [206]. In 2017, they used the triangular terpyridine-based metallocycles functionalized with 1-adamantyl and ferrocenyl groups as supramolecular cross-linkers for gelation of β-CD (β-cyclodextrine)-containing copolymers upon complexation with zinc (II) or iron (II) ions. The metallo-supramolecular gels were found responsive to multiple external stimuli, and the effect of the metal ions on gel–sol transformation was investigated. Specifically, the response to the destruction of the guest–host interactions through redox and dissociation reactions was explored. In addition, by adhesion tests the CPGs was found to be stimuli-responsive molecular glue to adhere the cross-linked gel blocks bearing β-CD.

In 2016 Shaoqin Gong and coworkers [207] exploited the unique properties possessed by carbon nanotubes (CNTs) to develop a Zn-CPG based on the metallo-supramolecular CNT/polyurethane interaction. A terpyridine ligand PU-terminated was in situ polymerizated on the surface of CNTs and subsequently crosslinked with the metal ion Zn^2+^. The nanocomposite shows interesting elastic mechanical properties, self-healing ability, and multiple stimuli responsiveness to NIR light, temperatures, and solvents, with short healing times. Another highly self-healing responsive CPG was examined very recently by Gengsheng Weng and coworkers [208], who used metal–alanine coordination as the dynamic cross-linker in a poly(N,N-dimethylacrylamide-co-3-alanine-2-hydroxypropylmethacrylate) network. By using different metal ions, several hydrogels of various rheological properties were obtained. Cu (II) and Zn (II)-assembled hydrogels work as the electrolytes and a Cu electrode as the cathode and a Zn electrode as the anode producing a self-powered hydrogel sensor. The probe is responsive to temperature, pH, chelator, and moisture.

## 5. Zn-CPs as Bioenginereed Tools for Theranostic Use

The unified theranostic platform offered by the new smart materials concerns sensing, drugs delivery for tissues treatment, and fluorescence bioimaging on cell samples. Design, synthesis, and applications of Zn-CPs in biomedicine are joint projects. In the next sections, the most relevant examples of Zn-CP bio-probes will be reviewed following a consequential and progressive approach. Specifically, we will refer to Zn-CPs designed for cellular sensing and drugs delivery and, finally, to Zn-NCPs for fluorescence bioimaging as highly engineered multifunctional tools for in vivo analysis.

### 5.1. Zn-LCPs for Sensing and Delivery of Bio-Relevant Species

Though traditional polymers are still dominant in drug delivery and bioimaging [209,210,211,212,213,214,215,216,217,218], they have some limits, such as the scarce water solubility which prevents interactions with the biological environment, and poor biodegradability which increases the biological risk. The hydrophilic–hydrophobic interactions offered by CPs and the dynamic invertibility of supramolecular interactions can be the key solving these problems. Specifically, the use of fluorescent CPs to detect and/or carry drugs such as anticancer, antibiotics, and anti-inflammatories is today’s challenge. In addition to tunable solubility and solvent-CP interaction, CPs can exhibit intrinsic biodegradability due to the relatively labile metal–ligand bond. The release of active molecules in biological substrates can be achieved by the responsiveness of the CPs probes to the environmental conditions. Absorption/desorption processes and ionic exchange can be obtained through biodegradation of the compound and alternatively by direct coordination of biomolecules to the polymeric substrate.

Zinc (II) cation is an essential element for life, non-toxic and even showing an antibacterial ability. Therefore, Zn-CPs can be biocompatible stimuli-responsive controlled-release materials for pharmaceutical applications. Several studies reported sensing of Zn-CPs for antibiotics and anti-inflammatories. In the most interesting articles, such ability marries drug carrier and release performances.

In 2016, Ana Belen Lago [219] and coworkers described a series of Zn-CPs prepared from 4-pyridylthio)methane with the anti-inflammatory ibuprofen incorporated as a ligand. The host materials exhibit high stability, good biodegradability and biocompatibility, and low cytotoxicity. The ibuprofen release process involves different ion exchange mechanisms depending on the coordination pattern of the probe. The drug release stages occur in a pH-controlled mode.

Multifunctional probes were recently developed by Jun Zhao and coworkers (see Figure 7) [220] which produced a 3D Zn-LCP based on 2,5-di(1H-1,2,4-triazol-1-yl) terephthalic frameworks with some active uncoordinated carboxylic groups, exhibiting luminescence and good water stability both in acidic and basic conditions (pH = 2–12). The probe acts as a promising dual functional sensor for detecting nitrofurazone (NFs), nitrofurantoin (NFT), and Fe^3+^ ions through fluorescence quenching mechanisms ascribed to a competitive absorption mechanism between the probe and the analytes.

Tests on in vivo real samples enrich and deepen some aspects of the therapeutic ability. Qinhe Pan and coworkers (see Figure 7) [221] in 2021 synthetised a Zn-LMOF (named HNU-55) from the organic ligand 3-pyridinesulfonic acid used as a fluorescent sensor for the detection of the antibiotic doxycycline (DOX) in real samples of pig feed. The fluorescent performance is effective in a wide linear range and with a low detection limit (3.7 nM). The mechanism of fluorescence enhancement was discussed based on the emission spectra and FT-IR analysis. Nanoscale zeolitic imidazolate framework-8 (ZIF-8) were employed by Wu and coworkers [222] in 2020 to build a cell-in-shell structure onto living cells, ablet to engineer the cell division by suppressing cell budding and growth. After the removal of the MOF shell, the cell growth could recover. Between the few examples of effective fabrication of nano-engineered biomedical devices, Yongan Wang and coworkers [223] fabricated a biocompatible and degradable Zn-MOF named ZIF-8@MeHA-MNs by encapsulating ZIF-8 into a crosslinked methacrylated hyaluronic acid (MeHA) hydrogel and molding the compound into microneedles (MNs). The MNs show antibacterial activity and steadily release of the active drugs in NIH-3T3 cell lines. Very recently, the antimicrobial and the antitumor activity of the Zn (II) complex with 3-(1-methyl-4-hydroxy-2-oxo-1,2-dihydroquinolin-3-yl)-2-nitro-3-oxopropanoic acid were evaluated against the Hepatocellular carcinoma cell line (HepG-2 cells) by Mosad A. El-ghamry and coworkers [224] and compared with analogous complexes from other transition metals.

Fengchun Hou [225] in 2020 designed a highly water soluble Zn-LCP [[Zn(DIPT)](NO3)(H2O)3] with the channel-type framework generated by the rigid three-terminal nitrogenous hetero-ligand 5-(3,5-di(1H-imidazol-1-yl)phenyl)-2H-tetrazole. The probe shows selective luminescence sensing for sulfonamide antibiotics and anti-bacterial activity against Porphuromonas gingivalis (P. gingivalis ATCC 33277) in vitro and in vivo. Finally, the in vivo inhibitory effect on periodontal tissue inflammation after orthodontics was tested by inhibiting the growth of Porphyromonas gingivalis. The inflammatory response by IL-8 and TNF-α level in gingival fluid was checked, and the binding patterns of the targeted protein NF-κB were indicated as the possible binding mode and regulation mechanism. Still in 2020, Mei Liu and coworkers [226] prepared a Zn-LMOF, namely [Zn(oba)2(bpy)2] (1) (where oba ¼ 4,40 -oxybisbenzoic acid, bpy ¼ 4,40—bipyridine) and used it as a sensitive and selective probe for a series of sulfonamides antibiotics and NACs via electron transfer process in aqueous solutions. The fluorescence quenching mechanism was not ascribed to the collapse of the framework but to an electron transfer or energy transfer process or a combination of the two phenomena between fluorophore and analytes. The probe was employed for the determination of sulfametahazine (SMZ) and 2,4,6-trinitrophenol (TNP) in synthetic urine and real wate samples.

Xia Zhou and coworkers [227] in 2020 produced an interesting study on a 3D Zn-CP based on a tris[4-(1H-1,2,4-triazol-1-yl)phenyl]amine, a 1,2,4-triazole and a flexible glutaric acid ligand able to give a luminescence quenching response to TNP. The polymer was investigated for the sepsis clinic treatment evaluating the anti-bacterial ability of the compound on the immune cells. The activation of the response in the macrophages under infection was checked revealing the detailed mechanism due to the upregulating of the miR-16, which is the negative regulator for the signalling pathway of NF-κB during an infection.

### 5.2. Multifunctional Zn-NCPs for Fluorescence Bioimaging

The terminology “imaging methods” refers to different techniques that are used for obtaining or producing images. In medical diagnostics, imaging methods give a view of an anatomical structure, check its functionality, and test the presence of any pathologies. In addition to morpho-structural information, imaging techniques can also provide functional data. For more than fifty years, medical imaging has been based on radiography and radioscopy. Only more recently, new technologies revolutionized this field with the introduction of ultrasound, nuclear medicine, and computed tomography (a computer assisted imaging technique, TAC). The nuclear medicine tomography techniques PET (Positron Emission Tomography), SPECT (Single Photon Emission Computed Tomography), magnetic resonance imaging (MRI), and even second-harmonic generation (SHG) were then introduced. These methods are currently considered to be of an advanced level (“advanced imaging”) [228,229,230,231,232,233,234,235,236].

Fluorescence bioimaging microscopy is among the most widely used modalities of live cell imaging. The success of some bioimaging techniques relies on the non-invasive nature and the ability to perform in-situ analysis both in vitro and in vivo [237]. To obtain effective bioimaging probes, low toxicity and deep tissue permeation properties must be added to the requirements of photochemical and metabolic stability and tuneable emissions [238,239,240,241,242]. Supramolecular materials have been recently used in fluorescence bioimaging, in addition to their sensing and drug-carrier uses.

Many efforts have been focused on the development of LCPs as an efficient alternative to the traditional polymeric probes. Due to the introduction of metal ions by metal coordination bonds, CPs can offer both the properties of the medal nodes and of the organic ligands to achieve optical and chemical properties employable in biological environments. Still retaining their original structural and chemical properties, CPs could serve as biological probes when scaled down to nanoscale coordination polymers (NCPs). The use of NCPs for bioimaging marries the most advantageous features of the stimuli-responsive CPs platform to the carrying/delivery properties of the nanoparticles, providing fluorescence microscopy probes with good cell penetration. Three-dimensional fluorescence imaging is not the only but surely the more relevant bioimaging technique, supported by the rapidly developing field of nanotechnology. Deeply penetrating nanosized probes, used to visualize the cellular compartments, the spatio-temporal dynamics of cells, and the tissue abnormalities, have been recently discussed in several excellent reviews [127,243,244]. NCPs range from quantum dots to carbon nanoparticles and LMOFs. Among the latter, various MOF probes were reported due to their unique structures and interaction ability.

Fluorescent Zn (II) complexes used as molecular probes for several bioimaging applications have been recently described [245] due to their simple and tuneable molecular structure, easy synthesis, and biocompatible nature. The outcomes of new techniques and methods developed in the last several years are derived from the biological prominence and the physiological significance of the zinc cation. The employ of zinc (II) metal nodes in building novel macromolecular tools for bioimaging meets the requirement of good cell penetration without perturbing the cell physiology. In addition, intrinsically biodegradable Zn-NCPs can produce easily expellable non-toxic fragments after the intended task is completed. Several stimuli-responsive Zn-NCPs, especially in the form of Zn-LMOFs, have been designed as a theranostic platform matching the biosensing ability, the therapeutical drugs delivery, and the bioimaging analysis. In this section, we will give a unified view of the progress in the last five years in the field of Zn-NCPs designed as multifunctional tools for living cell imaging techniques.

The use of nanoprobes based on biological ligands attracted the interest of researchers due to their scarce toxicity and biocompatibility. Peptide and DNA based NCPs showed great potential in biomedical applications. In 2017, Shukun Li and coworkers [246] used flexible short peptides in designing self-assembled materials for biomedical applications. They employed a histidine-containing dipeptide or an amphiphilic histidine derivative as the organic building block and zinc (II) cation for the self-assembly of colloidal NCPs apt to both drug release and fluorescence image of intracellular tumour microenvironments. In 2016, Mingjun Zhang and coworkers [247] produced a photostable and biocompatible Zn-NCPs from a tryptophan–phenylalanine dipeptide (Trp-Phe DNPs), emitting in the visible range. Trp-Phe DNPs was functionalized with the MUC1 aptamer, and doxorubicin was found to be a marker for cancer cells, employable as a combined tool in bioimaging and monitoring drug release. Very recently Jing Wei and coworkers synthesised water-dispersible CPs with different metal species (e.g., Gd, Cu, Ni, Zn, and Fe) using non-toxic plant polyphenol ligands (such as tannic acid and gallic acid) modified via a phenol–formaldehyde reaction. A competitive ligand was used to modulate the coordination assembly process to obtain colloidal NCPs highly water dispersible and permeable. The probes were used as a positive contrast agent for T1-weighted MR imaging of tumour cells. In 2020 Shengfeng Wang and coworkers [248] used ZIF-8 as building blocks for the synthesis of a DNA-based imaging nanoprobe. They designed a DNAzyme embedded molecular beacon (DMB) functionalizing ZIF-8 framework. ZIF-8 was disintegrated upon endocytosis releasing DMB for target mRNA detection and co-releasing zinc (II) cation as a cofactor to activate the embedded DNAzyme for mRNA regulation. Another nanoprobe for bioimaging based on ZIF-8 fragment was produced by T. Yu Tang and coworkers in 2017 [249] which synthetised a core-shell MOF-based nanocomposite UCNPs/MB@ZIF-8@ catalase (where UCNPs = upconverting nanoparticles, i.e., nanoscaled particles exhibiting photon upconversion of incident low energy photons into a higher energy emitted photon; MB = methylene blue; ZIF = zeolitic imidazolate framework) useful in bioimaging for selective monitoring of tumour cells.

With the aim of obtaining tailored therapeutic tools with combined ability in biosensing and bioimaging, several Zn-MOFs were recently proposed. Xiaoyan Liu and coworkers in 2018 [250] produced a Zn-MOF based on L = 2,5-bis(phenylamino)-1,4-benzenedicarboxylic acid named Zn_2_(L)_2_(DMF)_2_H_2_O exhibiting fluorescence enhancement for cadmium (II) ion and fluorescence quenching sensing in the presence of nitrobenzene well detectable in HepG2 cells bioimaging. A Zn-LMOF named [Zn_2_(OH)_2_(H_2_TCPP)](DMF)_3_] with 2,3,5,6-tetrakis (4-carboxyphenyl)pyrazine ligand selectively detecting picric acid and monitored for the inhibitory effect on various cancer cell lines (HeLa, CHO, HepG2, U251, MDA-MB-435 S, and BEAS-2B) was presented in 2020 by Hua Yang and coworkers [251].

**Figure 8:**
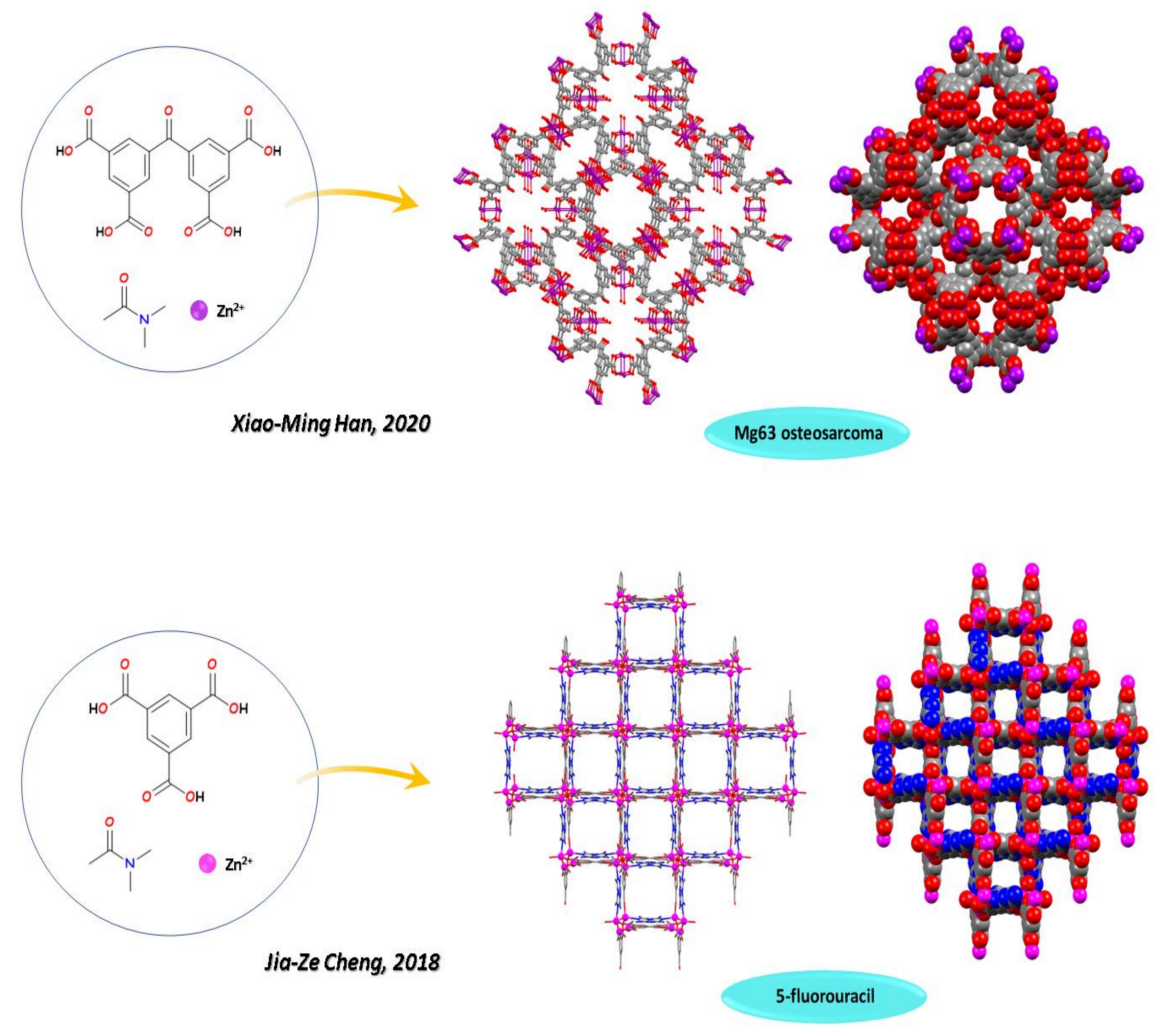
Some representative structures of Zn-CPs for fluorescence bioimaging. Figures readapted by using Mercury software [125].

Starting from 1D CPs, several fluorescence bioprobes with different functionality achieving a 3D supramolecular pattern have been designed. Xiaopeng Xuan and coworkers in 2019 produced a 1D homochiral Zn-CP starting from the achiral ligand 1,3-bis[2-(4-pyridyl)ethenyl]benzene. The infinite left (L)-handed helical chains are parallel to each other and further packing into a pseudo 3D network by weak C-H-Cl interactions leading to the crystallization in the tetragonal system P43212 chiral space group. Zn-NCPs with strong red fluorescence were employed in confocal fluorescence images of SH-SY5Ycells. Very recently, Chittaranjan Sinha and coworkers [252] synthesised two Zn-CPs of H2adc (Acetylene dicarboxylic acid)/trans-H2muca (trans, trans-muconic acid) along with capping 4-Cltpy (40-Chloro-2,20: 60, 200-terpyridine). Both probes are 1D CPs undergoing H-bonding and π–π interactions to form a 3D supramolecular network specifically detecting Cu^2+^ ions in aqueous media. Cell imaging was recorded to test the detection of intracellular Cu^2+^ within HepG2 cells. In the same year (2020), two Zn-LMOFs with highly specific catalytic activity were employed for cancer therapeutical treatment. Wei-Ping Zhang and coworkers [253] obtained from a rigid V-shaped ligand 2,6-di(2′,5′-dicarboxylphenyl)pyridine the Zn-LMOF named [Zn_3_(L)(OH)_2_(H_2_O)_4_](DMF)_5_] showing a catalytic activity for aromatic aldehydes cyanation and treatment ability on bladder cancer and Xiao-Ming Han and coworkers (see Figure 8) [254] produced a Zn-MOF named [Zn_2_(cipa) (H_2_O)_2_] (DMF)] from 5,5′-carbonyldiisophthalic acid with catalytic activity toward forming of C–C bonds of carbonyl compounds and valid for treatment against the Mg63 osteosarcoma cell line.

The application with the strongest biomedical and technological impact could be considered the drug-delivering in living cells. In the most desirable tools, the drug-delivery process can be monitored by real-time bioimaging techniques. The anticancer drug 5-fluorouracil (5-Fu) can be captured into the nanopores of the Zn-MOF named [Zn_3_(OH)_2_(H_2_tccp)_2_(bpy)_2_](H_2_O)_3_(DMF)_3_, from ligands 2,3,5,6-tetrakis(4-carboxyphenyl)pyrazine (H_4_tcpp) and 4,4′-bipyridine (bpy) (Suhui Wu and coworkers, 2020) and of the Zn-MOF named [Zn(BTC) (HME)](DMAc)(H_2_O), from ligands 1,3,5-benzenetricarboxylic acid (H_3_BTC) and HME = protonated melamine, DMAc = N,N-dimethylacetamide (Xiao-Tao Xin and Jia-Ze Cheng, 2018; see Figure 8 [255]). In both cases, the anti-proliferation activity was monitored by fluorescence bioimaging.

Highly bioengineered multifunctional probes were obtained by highly specific design of MOF-bioactive molecule blends. Very recently, Dong-Pyo Kim and coworkers [256] employed Janus carrier cells coated a Zn-MOF. Cytotoxic enzymes were encapsulated on the surface of carrier cells selectively releasable in presence of a chemotherapeutic protein of proteinase K in the cell environment. In 2017, Huwei Liu and coworkers [257] proposed the derivatization of surface acid groups of a Zn-MOF-COOH to give peptide linkages for immobilizing antibodies on the MOF. The functional modified probe combines cell recognition and capture with controllable drug delivery. Dongdong Sun and coworkers [258] in 2019 used gold nanoprisms (AuNPR) conjugated to tetraphenylethene (TPE)-functionalized phenanthroline and stabilized with target peptide aptamers via Au-S bonds (Au-Apt-TPE). The final product obtained by zinc coordination of the free nitrogen atoms (Au-Apt-TPE@Zn) was used for selectively monitoring early-stage apoptotic cells and showed deep penetration against SGC-7901 human gastric carcinoma cells growth in NIR bioimaging analysis. The probe Au-Apt-TPE@Zn was found to be an AIEgen. A special section of multifunctional bioimaging probes based on the use of fluorescence AIE (aggregation induced enhancement) active tools. AIE probes can solve problems due to fluorescence quenching of the aqueous aggregates in physiologic medium thanks to the strong emission in the concentred solution/aggregate state [105,259]. Another AIE zinc-based nanoprobe was developed in 2018 by Yun Yan and coworkers [260] as a fluorescent vesicle based on triarylamine carboxylate (TPA-1) forming the complex Zn(TPA-1)2, which further self-assembles into vesicles. The vesicles are cell imaging probes as tested by Hela cell imaging.

## 6. Conclusions

With the specific features of tuneable optical properties, tailored structures, and adsorption affinity, supramolecular CPs are widely utilized as polymeric smart materials. Specifically, their ability to change absorbance/emission spectral pattern in response to external stimuli (chromism) is a result of their unique hybrid nature and makes them excellent stimuli-responsive tools. In the search for new stimuli-responsive tools based on CP type structures, zinc (II) cation claims a role due to its non-toxic, abundant, and inexpensive “green soul” and to its highly versatile coordinative and structural ability. Moreover, its “optically innocent” ability to “clip” the ligands in emissive architectures makes it suitable to tailor chromogenic structures.

The present review summarizes the recent achievements of Zn-CPs in the role of stimuli-responsive materials providing a chromic response. An overview of the past five years (2016–2021) has been organised, encompassing 1, 2 and 3D (specifically MOFs and NCPs) responsive Zn-CPs. The most relevant examples of Zn-CPs were collected and discussed following a consequential and progressive approach. Specifically, we reviewed different Zn-CPs referring to their dimensionality, structure-responsiveness relationship, sensing mechanisms, and analytes and/or parameters detected. Stimuli-responsive Zn-CPs were reviewed as smart probes for the sensing of metal cations, oxyanions, and small and even harmful molecules; for the measurement of pH and temperature; as moulded in a soft-matter shape; as nanosized for the detection of biological targets, for drug release; and finally, for fluorescence bioimaging. In the last section of the review, a selection of cutting-edge articles about highly engineered Zn-CPs probes for advanced imaging technique was presented. The development of novel Zn-MOFs and Zn-NCPs capable of non-invasive cell permeation and drug-carriage was examined.

In summary, we provided an overview, which we hope will be a guide, of the novel chromogenic Zn-Cps by cutting across the large and complex topic of stimuli-responsive Cps.

## Data Availability

Data sharing not applicable.
